# Acetonyltri­phenyl­phospho­nium 2,3,5-tri­phenyl­tetra­zolium tetra­chlorido­cuprate(II)

**DOI:** 10.1107/S205698901701800X

**Published:** 2018-01-01

**Authors:** Mouhamadou Birame Diop, Libasse Diop, Allen G. Oliver

**Affiliations:** a Laboratoire de Chimie Minérale et Analytique, Département de Chimie, Faculté des Sciences et Techniques, Université Cheikh Anta Diop, Dakar, Senegal; b Department of Chemistry and Biochemistry, University of Notre Dame, 246, Nieuwland, Science Hall, Notre Dame, IN 46557-5670, USA

**Keywords:** crystal structure, acetonyltri­phenyl­phospho­nium cation, 2,3,5-tri­phenyl­tetra­zolium cation, tetra­chlorido­cuprate dianion, hydrogen bonds, three-dimensional structure

## Abstract

The title compound, (C_21_H_20_OP)(C_19_H_15_N_4_)[CuCl_4_], was obtained by reacting CuCl_2_·2H_2_O with a mixture of one equivalent of acetonyltri­phenyl­phospho­nium chloride and one equivalent of 2,3,5-tri­phenyl­tetra­zolium chloride in aceto­nitrile. In the structure, the Cu centre in the dianion is bonded to four chloride ligands and adopts a distorted tetra­hedral geometry. The phospho­nium cation likewise adopts the expected tetra­hedral geometry. The tetra­zolium ring forms dihedral angles of 77.68 (10), 26.85 (11) and 66.48 (10)° with the planes of the benzene rings of the substituent groups. In the crystal, weak C—H⋯Cl hydrogen-bonding inter­actions involving both cations and the anion give rise to a three-dimensional supra­molecular structure.

## Abstract 2

The product of the reaction of CuCl_2_·2H_2_O with both one equivalent of acetonyl tri­phenyl­phospho­nium chloride and one equivalent of 2,3,5-tri­phenyl­tetra­zolium chloride is described. The dianion, [CuCl_4_]^2−^, adopts a distorted tetra­hedral geometry.

## Chemical context   

Compounds containing the [CuCl_4_]^2−^ tetra­hedral dianion with various cations have been widely studied (Wei & Willett, 2002 ▸; Elangovan *et al.*, 2007 ▸; Haddad & Al-Far, 2008 ▸; Al-Ktaifani & Rukiah, 2012 ▸; Wikaira *et al.*, 2013 ▸; Laus *et al.*, 2015 ▸). Likewise, a few compounds with an acetonyl tri­phenyl­phospho­nium or 2,3,5-tri­phenyl­tetra­zolium cation have also been reported (Diop *et al.*, 2013 ▸, 2015 ▸; Zhang *et al.*, 2007 ▸). To expand on the available data on both the [CuCl_4_]^2−^ anion as well as that on acetonyltri­phenyl­phospho­nium and 2,3,5-tri­phenyl­tetra­zolium cations, we have initiated in this work the study of the inter­actions between CuCl_2_·2H_2_O, acetonyl tri­phenyl­phospho­nium chloride and 2,3,5-tri­phenyl­tetra­zolium chloride, expecting the presence of both cations in the resulting compound. This has yielded the title complex salt, (C_21_H_20_OP)^+^·(C_19_H_15_N_4_)^+^·[CuCl_4_]^2−^ whose crystal structure is reported herein.
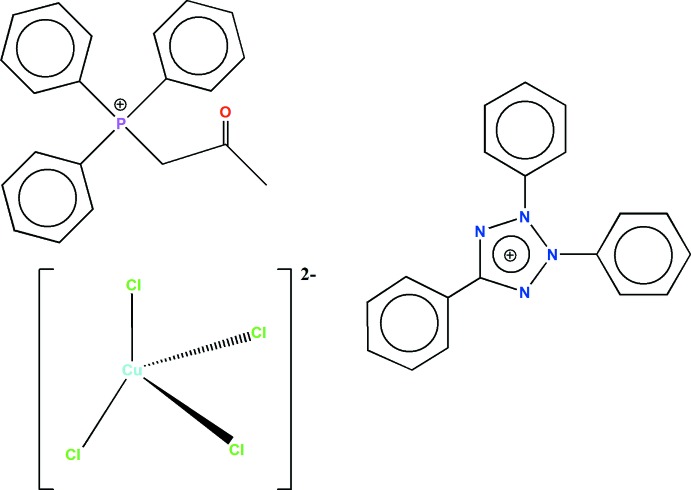


## Structural commentary   

**Figure 1:**
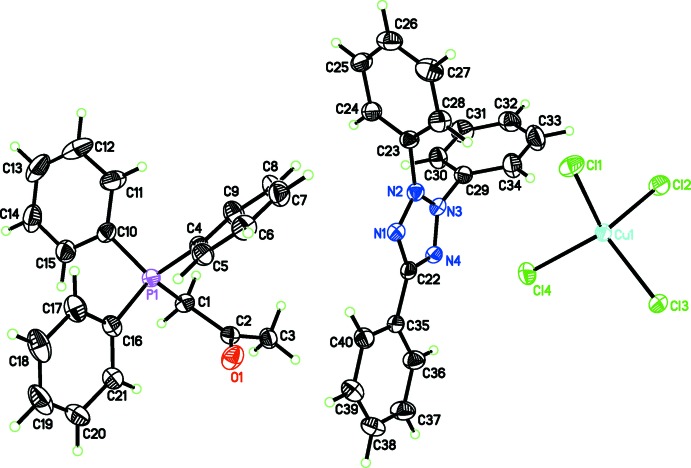
The mol­ecular components of the title compound. Displacement ellipsoids are drawn at the 50% probability level.

The asymmetric unit of the title complex comprises an acetonyl tri­phenyl­phospho­nium cation, a 2,3,5-tri­phenyl­tetra­zolium cation and a tetra­chlorido­cuprate dianion (Fig. 1 ▸). The environment around the Cu^II^ atom is distorted tetra­hedral with distances and angles [Cu—Cl = 2.2327 (6)–2.2540 (5) Å and Cl—Cu—Cl = 97.67 (2)–135.49 (2)°] in normal ranges for the [CuCl_4_]^2−^ complex anion (Clay *et al.*, 1975 ▸; Laus *et al.*, 2015 ▸). The P—C distances within the acetonyl tri­phenyl­phospho­nium cation are similar to those reported for the same cation (Diop *et al.*, 2013 ▸, 2015 ▸). The range for the C—P—C angles [107.07 (9)–113.36 (10)°] indicate a small variation of the geometry for this cation. Present in the cation is a C21—H⋯O1 inter­action [3.147 (3) Å with C—H⋯O angle = 115°]. The N—C and N—N distances within the 2,3,5-triphenyl tetra­zolium cation are consistent with a π delocalization in the tetra­zolium ring, which forms dihedral angles of 77.68 (10), 26.85 (11) and 66.48 (10)° with the planes of the benzene rings of the substituent groups.

## Supra­molecular features   

**Figure 2:**
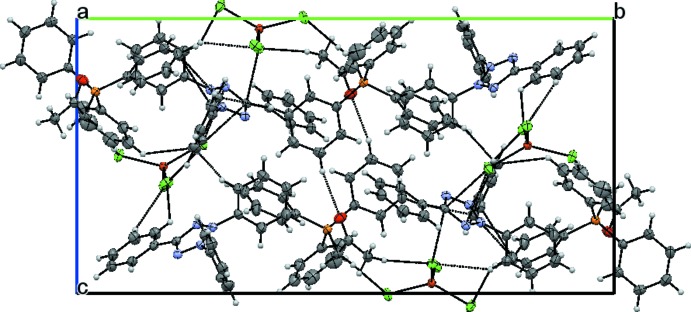
A view of the packing of the title compound viewed along [100], with hydrogen-bonding inter­actions shown as dashed lines. Displacement ellipsoids are drawn at the 50% probability level. The acetonyltriphenylphosphonium cations form supramolecular dimers through pairs of centrosymmetric C—H⋯O interactions.

In the crystal, inter-species C—H⋯Cl hydrogen bonds between aromatic, methyl­ene and methyl H atoms of the acetonyl tri­phenyl­phospho­nium cation and the [CuCl_4_]^2−^ anions are present (Table 1 ▸) together with weak C—H⋯Cl hydrogen-bonding inter­actions involving phenyl H atoms of the 2,3,5-triphenyl tetra­zolium cations. A three-dimensional supra­molecular structure is formed (Fig. 2 ▸).

## Database survey   

A search of the Cambridge Structural Database (CSD version 5.39; Groom *et al.*, 2016 ▸) returned hundreds and hundreds of different structures containing the [CuCl_4_]^2−^ dianion. To date, only nine structures of acetonyl tri­phenyl­phospho­nium and seventeen structures of 2,3,5-tri­phenyl­tetra­zolium have been deposited in the CSD. No structure including both acetonyltri­phenyl­phospho­nium and 2,3,5-tri­phenyl­tetra­zolium species was found.

## Synthesis and crystallization   

All chemicals were purchased from Aldrich Company, Germany and used as received. Acetonyl tri­phenyl­phospho­nium chloride and 2,3,5-triphenyl tetra­zolium chloride were mixed in aceto­nitrile with CuCl_2_·2H_2_O in a 1:1:1 ratio: a yellow–orange solution was obtained. Orange crystals suitable for a single-crystal X-ray diffraction study were obtained after a slow solvent evaporation at room temperature (300 K).

## Refinement   

Crystal data, data collection and structure refinement details are summarized in Table 2 ▸. All H atoms were placed at calculated positions and refined as riding atoms, with C—H = 0.95 Å (aromatic), 0.99 Å (methyl­ene) or 0.98 Å (meth­yl), and with *U*_iso_(H) = 1.2*U*_eq_(aromatic or methyl­ene) or 1.5*U*_eq_(meth­yl).

## Supplementary Material

Crystal structure: contains datablock(s) I. DOI: 10.1107/S205698901701800X/zs2394sup1.cif

Structure factors: contains datablock(s) I. DOI: 10.1107/S205698901701800X/zs2394Isup2.hkl

CCDC reference: 1811873

Additional supporting information:  crystallographic information; 3D view; checkCIF report

## Figures and Tables

**Table 1:** Hydrogen-bond geometry (Å, °)

*D*—H⋯*A*	*D*—H	H⋯*A*	*D*⋯*A*	*D*—H⋯*A*
C1—H1*A*⋯Cl2^i^	0.99	2.54	3.363 (2)	141
C1—H1*B*⋯Cl2^ii^	0.99	2.69	3.662 (2)	168
C3—H3*B*⋯Cl3^ii^	0.98	2.74	3.714 (2)	171
C3—H3*C*⋯Cl2^i^	0.98	2.90	3.630 (2)	132
C9—H9⋯Cl2^ii^	0.95	2.97	3.917 (2)	172
C24—H24⋯Cl3^ii^	0.95	2.99	3.783 (2)	142
C28—H28⋯Cl1	0.95	2.72	3.605 (2)	156
C30—H30⋯Cl3^ii^	0.95	2.87	3.683 (2)	144
C30—H30⋯Cl4^ii^	0.95	2.85	3.630 (2)	140

Symmetry codes: (i) 
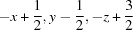
; (ii) 
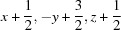
.

**Table 2:** Experimental details

Crystal data	
Chemical formula	(C_21_H_20_OP)(C_19_H_15_N_4_)[CuCl_4_]
*M*_r_	824.03
Crystal system, space group	Monoclinic, *P*2_1_/*n*
Temperature (K)	120
*a*, *b*, *c* (Å)	10.6868 (12), 26.421 (3), 13.5628 (15)
β (°)	90.709 (1)
*V* (Å^3^)	3829.2 (7)
*Z*	4
Radiation type	Mo *K*α
μ (mm^−1^)	0.93
Crystal size (mm)	0.29 × 0.20 × 0.16

Data collection	
Diffractometer	Bruker APEXII
Absorption correction	Numerical (*SADABS*; Krause *et al.*, 2015 ▸)
*T*_min_, *T*_max_	0.850, 0.939
No. of measured, independent and observed [*I* > 2σ(*I*)] reflections	86744, 9517, 7659
*R*_int_	0.046
(sin θ/λ)_max_ (Å^−1^)	0.668

Refinement	
*R*[*F* ^2^ > 2σ(*F* ^2^)], *wR*(*F* ^2^), *S*	0.037, 0.096, 1.04
No. of reflections	9517
No. of parameters	461
H-atom treatment	H-atom parameters constrained
Δρ_max_, Δρ_min_ (e Å^−3^)	0.59, −0.31

Computer programs: *APEX2* , *SAINT* and *XP* (Bruker, 2015 ▸), *SHELXS2014* (Sheldrick, 2015*a* ▸), *SHELXL2014* (Sheldrick, 2015*b* ▸) and *CIFTAB* (Sheldrick, 2015*b* ▸).

## supplementary crystallographic information

### Crystal data

**Table:**

(C_21_H_20_OP)(C_19_H_15_N_4_)[CuCl_4_]	*F*(000) = 1692
*M**_r_* = 824.03	*D*_x_ = 1.429 Mg m^−^^3^
Monoclinic, *P*2_1_/*n*	Mo *K*α radiation, λ = 0.71073 Å
*a* = 10.6868 (12) Å	Cell parameters from 9465 reflections
*b* = 26.421 (3) Å	θ = 2.4–28.1°
*c* = 13.5628 (15) Å	µ = 0.93 mm^−^^1^
β = 90.709 (1)°	*T* = 120 K
*V* = 3829.2 (7) Å^3^	Irregular fragment, orange
*Z* = 4	0.29 × 0.20 × 0.16 mm

### Data collection

**Table:**

Bruker APEXII diffractometer	9517 independent reflections
Radiation source: fine-focus sealed tube	7659 reflections with *I* > 2σ(*I*)
Graphite monochromator	*R*_int_ = 0.046
Detector resolution: 8.33 pixels mm^-1^	θ_max_ = 28.3°, θ_min_ = 1.5°
combination of ω and φ–scans	*h* = −14→14
Absorption correction: numerical (SADABS; Krause *et al.*, 2015)	*k* = −35→35
*T*_min_ = 0.850, *T*_max_ = 0.939	*l* = −18→18
86744 measured reflections	

### Refinement

**Table:**

Refinement on *F*^2^	Primary atom site location: real-space vector search
Least-squares matrix: full	Secondary atom site location: difference Fourier map
*R*[*F*^2^ > 2σ(*F*^2^)] = 0.037	Hydrogen site location: inferred from neighbouring sites
*wR*(*F*^2^) = 0.096	H-atom parameters constrained
*S* = 1.04	*w* = 1/[σ^2^(*F*_o_^2^) + (0.0444*P*)^2^ + 2.8241*P*] where *P* = (*F*_o_^2^ + 2*F*_c_^2^)/3
9517 reflections	(Δ/σ)_max_ = 0.003
461 parameters	Δρ_max_ = 0.59 e Å^−^^3^
0 restraints	Δρ_min_ = −0.31 e Å^−^^3^

### Special details

**Table:**

Geometry. All esds (except the esd in the dihedral angle between two l.s. planes) are estimated using the full covariance matrix. The cell esds are taken into account individually in the estimation of esds in distances, angles and torsion angles; correlations between esds in cell parameters are only used when they are defined by crystal symmetry. An approximate (isotropic) treatment of cell esds is used for estimating esds involving l.s. planes.

### Fractional atomic coordinates and isotropic or equivalent isotropic displacement parameters (Å^2^)

**Table:**

	*x*	*y*	*z*	*U*_iso_*/*U*_eq_	
Cu1	0.18416 (2)	0.83950 (2)	0.46171 (2)	0.01990 (7)	
Cl1	0.37755 (5)	0.83273 (2)	0.40187 (4)	0.03219 (12)	
Cl2	0.18895 (5)	0.92209 (2)	0.50256 (4)	0.03061 (12)	
Cl3	−0.00778 (4)	0.83854 (2)	0.39095 (4)	0.02588 (11)	
Cl4	0.17809 (5)	0.76656 (2)	0.54465 (4)	0.03418 (13)	
P1	0.62501 (5)	0.46610 (2)	0.76661 (4)	0.02193 (11)	
O1	0.35961 (15)	0.49029 (6)	0.72476 (11)	0.0365 (4)	
C1	0.51075 (18)	0.48642 (8)	0.85438 (14)	0.0242 (4)	
H1A	0.4910	0.4578	0.8987	0.029*	
H1B	0.5471	0.5139	0.8953	0.029*	
C2	0.38982 (18)	0.50531 (8)	0.80594 (15)	0.0258 (4)	
C3	0.3142 (2)	0.54137 (8)	0.86465 (16)	0.0299 (4)	
H3A	0.2388	0.5508	0.8271	0.045*	
H3B	0.3639	0.5718	0.8788	0.045*	
H3C	0.2902	0.5253	0.9267	0.045*	
C4	0.64250 (18)	0.51654 (8)	0.67895 (15)	0.0242 (4)	
C5	0.6371 (2)	0.50811 (8)	0.57820 (15)	0.0286 (4)	
H5	0.6253	0.4748	0.5534	0.034*	
C6	0.6490 (2)	0.54866 (9)	0.51329 (16)	0.0344 (5)	
H6	0.6462	0.5430	0.4441	0.041*	
C7	0.6650 (2)	0.59706 (9)	0.54977 (18)	0.0363 (5)	
H7	0.6730	0.6248	0.5056	0.044*	
C8	0.6693 (2)	0.60526 (9)	0.65035 (18)	0.0372 (5)	
H8	0.6805	0.6386	0.6749	0.045*	
C9	0.6576 (2)	0.56546 (8)	0.71550 (17)	0.0333 (5)	
H9	0.6599	0.5714	0.7846	0.040*	
C10	0.76907 (18)	0.45595 (8)	0.83306 (15)	0.0262 (4)	
C11	0.8699 (2)	0.48849 (10)	0.82425 (19)	0.0408 (6)	
H11	0.8650	0.5172	0.7822	0.049*	
C12	0.9787 (2)	0.47832 (13)	0.8783 (2)	0.0576 (8)	
H12	1.0487	0.5002	0.8725	0.069*	
C13	0.9863 (2)	0.43719 (12)	0.9400 (2)	0.0528 (7)	
H13	1.0613	0.4307	0.9760	0.063*	
C14	0.8858 (2)	0.40559 (10)	0.9494 (2)	0.0450 (6)	
H14	0.8908	0.3775	0.9930	0.054*	
C15	0.7763 (2)	0.41431 (9)	0.89564 (18)	0.0361 (5)	
H15	0.7070	0.3920	0.9016	0.043*	
C16	0.5878 (2)	0.40659 (8)	0.71009 (15)	0.0268 (4)	
C17	0.6791 (2)	0.38438 (9)	0.65135 (16)	0.0355 (5)	
H17	0.7554	0.4017	0.6396	0.043*	
C18	0.6582 (3)	0.33723 (9)	0.61035 (18)	0.0441 (6)	
H18	0.7192	0.3223	0.5691	0.053*	
C19	0.5484 (3)	0.31196 (9)	0.6296 (2)	0.0500 (7)	
H19	0.5346	0.2793	0.6022	0.060*	
C20	0.4587 (3)	0.33336 (9)	0.6881 (2)	0.0486 (7)	
H20	0.3836	0.3154	0.7008	0.058*	
C21	0.4772 (2)	0.38109 (8)	0.72871 (18)	0.0355 (5)	
H21	0.4148	0.3961	0.7687	0.043*	
N1	0.41588 (15)	0.68767 (6)	0.64421 (12)	0.0231 (3)	
N2	0.46486 (15)	0.72999 (6)	0.67689 (12)	0.0218 (3)	
N3	0.38773 (14)	0.75180 (6)	0.74104 (12)	0.0219 (3)	
N4	0.28719 (15)	0.72419 (6)	0.75175 (12)	0.0225 (3)	
C22	0.30628 (17)	0.68472 (7)	0.69106 (14)	0.0217 (4)	
C23	0.58844 (18)	0.74780 (7)	0.65044 (15)	0.0237 (4)	
C24	0.68680 (19)	0.73834 (8)	0.71430 (16)	0.0280 (4)	
H24	0.6733	0.7241	0.7776	0.034*	
C25	0.80653 (19)	0.75032 (8)	0.68297 (17)	0.0318 (5)	
H25	0.8765	0.7440	0.7251	0.038*	
C26	0.8245 (2)	0.77132 (9)	0.59150 (18)	0.0349 (5)	
H26	0.9067	0.7793	0.5707	0.042*	
C27	0.7234 (2)	0.78090 (10)	0.52971 (17)	0.0389 (5)	
H27	0.7367	0.7956	0.4668	0.047*	
C28	0.6029 (2)	0.76927 (9)	0.55851 (16)	0.0315 (5)	
H28	0.5329	0.7758	0.5166	0.038*	
C29	0.40967 (18)	0.80011 (8)	0.78841 (15)	0.0243 (4)	
C30	0.4634 (2)	0.80027 (8)	0.88116 (16)	0.0315 (5)	
H30	0.4889	0.7697	0.9122	0.038*	
C31	0.4789 (2)	0.84658 (9)	0.92767 (18)	0.0392 (5)	
H31	0.5162	0.8481	0.9915	0.047*	
C32	0.4403 (2)	0.89058 (9)	0.88143 (19)	0.0376 (5)	
H32	0.4505	0.9221	0.9141	0.045*	
C33	0.3869 (3)	0.88917 (9)	0.7881 (2)	0.0442 (6)	
H33	0.3608	0.9197	0.7570	0.053*	
C34	0.3714 (2)	0.84312 (8)	0.73976 (18)	0.0373 (5)	
H34	0.3356	0.8415	0.6754	0.045*	
C35	0.21638 (18)	0.64331 (7)	0.67518 (14)	0.0225 (4)	
C36	0.0992 (2)	0.64553 (9)	0.71863 (17)	0.0314 (5)	
H36	0.0781	0.6733	0.7596	0.038*	
C37	0.0135 (2)	0.60693 (9)	0.70173 (18)	0.0371 (5)	
H37	−0.0665	0.6081	0.7314	0.045*	
C38	0.0449 (2)	0.56676 (9)	0.64165 (17)	0.0344 (5)	
H38	−0.0146	0.5408	0.6287	0.041*	
C39	0.1622 (2)	0.56409 (8)	0.60035 (16)	0.0322 (5)	
H39	0.1837	0.5359	0.5605	0.039*	
C40	0.24869 (19)	0.60236 (8)	0.61681 (15)	0.0276 (4)	
H40	0.3294	0.6005	0.5884	0.033*	

### Atomic displacement parameters (Å^2^)

**Table:**

	*U*^11^	*U*^22^	*U*^33^	*U*^12^	*U*^13^	*U*^23^
Cu1	0.01889 (12)	0.01888 (12)	0.02188 (12)	0.00136 (8)	−0.00154 (8)	−0.00022 (9)
Cl1	0.0206 (2)	0.0399 (3)	0.0361 (3)	0.0023 (2)	0.00368 (19)	−0.0073 (2)
Cl2	0.0262 (2)	0.0225 (2)	0.0430 (3)	0.00192 (19)	−0.0028 (2)	−0.0096 (2)
Cl3	0.0214 (2)	0.0246 (2)	0.0315 (2)	−0.00047 (17)	−0.00662 (18)	0.00112 (19)
Cl4	0.0374 (3)	0.0270 (3)	0.0379 (3)	−0.0007 (2)	−0.0100 (2)	0.0112 (2)
P1	0.0215 (2)	0.0225 (2)	0.0219 (2)	−0.00009 (19)	0.00078 (18)	−0.00152 (19)
O1	0.0346 (8)	0.0422 (9)	0.0324 (8)	0.0075 (7)	−0.0091 (7)	−0.0084 (7)
C1	0.0229 (9)	0.0283 (10)	0.0214 (9)	0.0004 (8)	0.0029 (7)	−0.0025 (8)
C2	0.0239 (9)	0.0245 (10)	0.0290 (10)	−0.0021 (8)	0.0016 (8)	0.0005 (8)
C3	0.0303 (11)	0.0227 (10)	0.0366 (11)	0.0029 (8)	0.0051 (9)	0.0017 (9)
C4	0.0238 (9)	0.0230 (10)	0.0260 (10)	0.0013 (7)	0.0034 (7)	−0.0004 (8)
C5	0.0312 (11)	0.0266 (11)	0.0279 (10)	−0.0002 (8)	0.0005 (8)	−0.0029 (8)
C6	0.0367 (12)	0.0402 (13)	0.0263 (11)	−0.0003 (10)	0.0013 (9)	0.0045 (9)
C7	0.0385 (12)	0.0321 (12)	0.0385 (12)	0.0040 (10)	0.0016 (10)	0.0100 (10)
C8	0.0474 (14)	0.0222 (11)	0.0422 (13)	0.0008 (10)	0.0035 (10)	0.0010 (9)
C9	0.0450 (13)	0.0259 (11)	0.0293 (11)	0.0020 (9)	0.0020 (9)	−0.0029 (9)
C10	0.0230 (9)	0.0288 (11)	0.0268 (10)	0.0033 (8)	0.0011 (7)	−0.0032 (8)
C11	0.0267 (11)	0.0484 (15)	0.0471 (14)	−0.0069 (10)	−0.0003 (10)	0.0021 (11)
C12	0.0228 (12)	0.078 (2)	0.072 (2)	−0.0072 (13)	−0.0036 (12)	−0.0031 (17)
C13	0.0309 (13)	0.0672 (19)	0.0598 (17)	0.0169 (13)	−0.0146 (12)	−0.0122 (15)
C14	0.0468 (15)	0.0390 (14)	0.0488 (15)	0.0165 (11)	−0.0157 (12)	−0.0051 (11)
C15	0.0357 (12)	0.0309 (12)	0.0414 (13)	0.0038 (9)	−0.0095 (10)	−0.0019 (10)
C16	0.0335 (11)	0.0218 (10)	0.0249 (10)	−0.0004 (8)	−0.0032 (8)	0.0005 (8)
C17	0.0474 (13)	0.0300 (12)	0.0292 (11)	0.0033 (10)	0.0042 (10)	−0.0011 (9)
C18	0.0739 (19)	0.0283 (12)	0.0300 (12)	0.0084 (12)	0.0016 (12)	−0.0045 (9)
C19	0.078 (2)	0.0225 (12)	0.0493 (16)	−0.0013 (12)	−0.0186 (14)	−0.0061 (11)
C20	0.0479 (15)	0.0266 (12)	0.0709 (19)	−0.0099 (11)	−0.0150 (14)	−0.0003 (12)
C21	0.0342 (12)	0.0270 (11)	0.0452 (13)	−0.0009 (9)	−0.0055 (10)	0.0008 (10)
N1	0.0213 (8)	0.0232 (8)	0.0247 (8)	0.0005 (6)	−0.0019 (6)	0.0004 (6)
N2	0.0208 (8)	0.0220 (8)	0.0225 (8)	0.0013 (6)	0.0011 (6)	−0.0002 (6)
N3	0.0190 (7)	0.0237 (8)	0.0228 (8)	0.0030 (6)	−0.0006 (6)	0.0000 (6)
N4	0.0199 (8)	0.0236 (8)	0.0240 (8)	−0.0001 (6)	−0.0017 (6)	0.0026 (6)
C22	0.0205 (9)	0.0227 (9)	0.0219 (9)	0.0018 (7)	−0.0015 (7)	0.0039 (7)
C23	0.0207 (9)	0.0227 (10)	0.0277 (10)	−0.0007 (7)	0.0031 (7)	−0.0026 (8)
C24	0.0261 (10)	0.0290 (11)	0.0288 (10)	−0.0021 (8)	−0.0004 (8)	0.0015 (8)
C25	0.0226 (10)	0.0316 (11)	0.0411 (12)	−0.0017 (8)	−0.0005 (8)	−0.0031 (9)
C26	0.0296 (11)	0.0316 (12)	0.0438 (13)	−0.0066 (9)	0.0111 (9)	−0.0080 (10)
C27	0.0420 (13)	0.0451 (14)	0.0300 (11)	−0.0077 (11)	0.0124 (10)	0.0028 (10)
C28	0.0318 (11)	0.0367 (12)	0.0260 (10)	−0.0025 (9)	−0.0008 (8)	0.0019 (9)
C29	0.0227 (9)	0.0232 (10)	0.0270 (10)	0.0004 (7)	0.0027 (7)	−0.0026 (8)
C30	0.0374 (12)	0.0273 (11)	0.0298 (11)	0.0035 (9)	−0.0021 (9)	−0.0011 (9)
C31	0.0462 (14)	0.0373 (13)	0.0339 (12)	−0.0017 (11)	−0.0022 (10)	−0.0083 (10)
C32	0.0379 (12)	0.0277 (11)	0.0472 (14)	−0.0023 (9)	0.0065 (10)	−0.0092 (10)
C33	0.0549 (16)	0.0244 (12)	0.0531 (15)	0.0043 (11)	−0.0057 (12)	0.0016 (11)
C34	0.0471 (14)	0.0284 (12)	0.0361 (12)	0.0035 (10)	−0.0086 (10)	0.0017 (9)
C35	0.0215 (9)	0.0220 (9)	0.0239 (9)	−0.0004 (7)	−0.0022 (7)	0.0033 (7)
C36	0.0251 (10)	0.0324 (11)	0.0368 (12)	−0.0023 (9)	0.0049 (8)	−0.0037 (9)
C37	0.0284 (11)	0.0386 (13)	0.0446 (13)	−0.0076 (9)	0.0072 (9)	−0.0040 (10)
C38	0.0356 (12)	0.0298 (11)	0.0379 (12)	−0.0109 (9)	0.0009 (9)	0.0005 (9)
C39	0.0391 (12)	0.0257 (11)	0.0320 (11)	−0.0035 (9)	0.0021 (9)	−0.0016 (9)
C40	0.0275 (10)	0.0256 (10)	0.0299 (10)	0.0009 (8)	0.0047 (8)	0.0013 (8)

### Geometric parameters (Å, º)

**Table:**

Cu1—Cl4	2.2327 (6)	C19—H19	0.9500
Cu1—Cl1	2.2368 (6)	C20—C21	1.389 (3)
Cu1—Cl2	2.2518 (6)	C20—H20	0.9500
Cu1—Cl3	2.2540 (5)	C21—H21	0.9500
P1—C16	1.792 (2)	N1—N2	1.310 (2)
P1—C10	1.794 (2)	N1—C22	1.342 (2)
P1—C4	1.797 (2)	N2—N3	1.336 (2)
P1—C1	1.7979 (19)	N2—C23	1.451 (2)
O1—C2	1.210 (3)	N3—N4	1.308 (2)
C1—C2	1.526 (3)	N3—C29	1.447 (3)
C1—H1A	0.9900	N4—C22	1.346 (3)
C1—H1B	0.9900	C22—C35	1.470 (3)
C2—C3	1.487 (3)	C23—C24	1.377 (3)
C3—H3A	0.9800	C23—C28	1.380 (3)
C3—H3B	0.9800	C24—C25	1.390 (3)
C3—H3C	0.9800	C24—H24	0.9500
C4—C5	1.385 (3)	C25—C26	1.375 (3)
C4—C9	1.393 (3)	C25—H25	0.9500
C5—C6	1.393 (3)	C26—C27	1.383 (3)
C5—H5	0.9500	C26—H26	0.9500
C6—C7	1.381 (3)	C27—C28	1.384 (3)
C6—H6	0.9500	C27—H27	0.9500
C7—C8	1.381 (3)	C28—H28	0.9500
C7—H7	0.9500	C29—C34	1.374 (3)
C8—C9	1.380 (3)	C29—C30	1.376 (3)
C8—H8	0.9500	C30—C31	1.385 (3)
C9—H9	0.9500	C30—H30	0.9500
C10—C11	1.385 (3)	C31—C32	1.382 (3)
C10—C15	1.391 (3)	C31—H31	0.9500
C11—C12	1.393 (4)	C32—C33	1.383 (4)
C11—H11	0.9500	C32—H32	0.9500
C12—C13	1.373 (4)	C33—C34	1.391 (3)
C12—H12	0.9500	C33—H33	0.9500
C13—C14	1.368 (4)	C34—H34	0.9500
C13—H13	0.9500	C35—C40	1.387 (3)
C14—C15	1.390 (3)	C35—C36	1.391 (3)
C14—H14	0.9500	C36—C37	1.388 (3)
C15—H15	0.9500	C36—H36	0.9500
C16—C21	1.387 (3)	C37—C38	1.382 (3)
C16—C17	1.396 (3)	C37—H37	0.9500
C17—C18	1.381 (3)	C38—C39	1.381 (3)
C17—H17	0.9500	C38—H38	0.9500
C18—C19	1.378 (4)	C39—C40	1.386 (3)
C18—H18	0.9500	C39—H39	0.9500
C19—C20	1.373 (4)	C40—H40	0.9500
			
Cl4—Cu1—Cl1	98.43 (2)	C20—C19—H19	119.6
Cl4—Cu1—Cl2	135.49 (2)	C18—C19—H19	119.6
Cl1—Cu1—Cl2	98.54 (2)	C19—C20—C21	120.4 (3)
Cl4—Cu1—Cl3	99.95 (2)	C19—C20—H20	119.8
Cl1—Cu1—Cl3	133.21 (2)	C21—C20—H20	119.8
Cl2—Cu1—Cl3	97.67 (2)	C16—C21—C20	119.0 (2)
C16—P1—C10	105.55 (10)	C16—C21—H21	120.5
C16—P1—C4	113.08 (9)	C20—C21—H21	120.5
C10—P1—C4	110.33 (10)	N2—N1—C22	103.71 (16)
C16—P1—C1	113.36 (10)	N1—N2—N3	109.96 (15)
C10—P1—C1	107.31 (9)	N1—N2—C23	123.66 (16)
C4—P1—C1	107.07 (9)	N3—N2—C23	126.28 (16)
C2—C1—P1	113.02 (14)	N4—N3—N2	110.24 (15)
C2—C1—H1A	109.0	N4—N3—C29	124.84 (16)
P1—C1—H1A	109.0	N2—N3—C29	124.86 (16)
C2—C1—H1B	109.0	N3—N4—C22	103.49 (15)
P1—C1—H1B	109.0	N1—C22—N4	112.61 (17)
H1A—C1—H1B	107.8	N1—C22—C35	123.14 (18)
O1—C2—C3	123.83 (19)	N4—C22—C35	124.23 (17)
O1—C2—C1	119.95 (18)	C24—C23—C28	123.34 (19)
C3—C2—C1	116.22 (17)	C24—C23—N2	118.41 (18)
C2—C3—H3A	109.5	C28—C23—N2	117.98 (18)
C2—C3—H3B	109.5	C23—C24—C25	117.7 (2)
H3A—C3—H3B	109.5	C23—C24—H24	121.1
C2—C3—H3C	109.5	C25—C24—H24	121.1
H3A—C3—H3C	109.5	C26—C25—C24	120.5 (2)
H3B—C3—H3C	109.5	C26—C25—H25	119.8
C5—C4—C9	120.24 (19)	C24—C25—H25	119.8
C5—C4—P1	122.00 (16)	C25—C26—C27	120.3 (2)
C9—C4—P1	117.72 (15)	C25—C26—H26	119.9
C4—C5—C6	119.8 (2)	C27—C26—H26	119.9
C4—C5—H5	120.1	C26—C27—C28	120.7 (2)
C6—C5—H5	120.1	C26—C27—H27	119.6
C7—C6—C5	119.8 (2)	C28—C27—H27	119.6
C7—C6—H6	120.1	C23—C28—C27	117.5 (2)
C5—C6—H6	120.1	C23—C28—H28	121.3
C6—C7—C8	120.1 (2)	C27—C28—H28	121.3
C6—C7—H7	120.0	C34—C29—C30	123.7 (2)
C8—C7—H7	120.0	C34—C29—N3	118.08 (18)
C9—C8—C7	120.7 (2)	C30—C29—N3	118.17 (18)
C9—C8—H8	119.6	C29—C30—C31	117.7 (2)
C7—C8—H8	119.6	C29—C30—H30	121.1
C8—C9—C4	119.3 (2)	C31—C30—H30	121.1
C8—C9—H9	120.3	C32—C31—C30	120.2 (2)
C4—C9—H9	120.3	C32—C31—H31	119.9
C11—C10—C15	120.4 (2)	C30—C31—H31	119.9
C11—C10—P1	121.84 (17)	C31—C32—C33	120.7 (2)
C15—C10—P1	117.74 (16)	C31—C32—H32	119.7
C10—C11—C12	118.7 (3)	C33—C32—H32	119.7
C10—C11—H11	120.7	C32—C33—C34	120.0 (2)
C12—C11—H11	120.7	C32—C33—H33	120.0
C13—C12—C11	121.1 (3)	C34—C33—H33	120.0
C13—C12—H12	119.5	C29—C34—C33	117.6 (2)
C11—C12—H12	119.5	C29—C34—H34	121.2
C14—C13—C12	120.0 (2)	C33—C34—H34	121.2
C14—C13—H13	120.0	C40—C35—C36	120.41 (19)
C12—C13—H13	120.0	C40—C35—C22	119.82 (18)
C13—C14—C15	120.4 (3)	C36—C35—C22	119.77 (18)
C13—C14—H14	119.8	C37—C36—C35	119.6 (2)
C15—C14—H14	119.8	C37—C36—H36	120.2
C14—C15—C10	119.4 (2)	C35—C36—H36	120.2
C14—C15—H15	120.3	C38—C37—C36	119.8 (2)
C10—C15—H15	120.3	C38—C37—H37	120.1
C21—C16—C17	120.2 (2)	C36—C37—H37	120.1
C21—C16—P1	122.23 (17)	C39—C38—C37	120.4 (2)
C17—C16—P1	117.39 (17)	C39—C38—H38	119.8
C18—C17—C16	119.9 (2)	C37—C38—H38	119.8
C18—C17—H17	120.1	C38—C39—C40	120.3 (2)
C16—C17—H17	120.1	C38—C39—H39	119.9
C19—C18—C17	119.6 (3)	C40—C39—H39	119.9
C19—C18—H18	120.2	C39—C40—C35	119.39 (19)
C17—C18—H18	120.2	C39—C40—H40	120.3
C20—C19—C18	120.8 (2)	C35—C40—H40	120.3
			
C16—P1—C1—C2	−73.90 (17)	C22—N1—N2—C23	176.44 (17)
C10—P1—C1—C2	169.96 (14)	N1—N2—N3—N4	0.2 (2)
C4—P1—C1—C2	51.51 (17)	C23—N2—N3—N4	−176.19 (17)
P1—C1—C2—O1	25.5 (3)	N1—N2—N3—C29	−177.26 (16)
P1—C1—C2—C3	−155.48 (15)	C23—N2—N3—C29	6.4 (3)
C16—P1—C4—C5	−4.8 (2)	N2—N3—N4—C22	−0.21 (19)
C10—P1—C4—C5	113.14 (18)	C29—N3—N4—C22	177.20 (17)
C1—P1—C4—C5	−130.39 (17)	N2—N1—C22—N4	−0.1 (2)
C16—P1—C4—C9	173.01 (17)	N2—N1—C22—C35	178.44 (17)
C10—P1—C4—C9	−69.04 (19)	N3—N4—C22—N1	0.2 (2)
C1—P1—C4—C9	47.43 (19)	N3—N4—C22—C35	−178.33 (17)
C9—C4—C5—C6	1.1 (3)	N1—N2—C23—C24	−97.3 (2)
P1—C4—C5—C6	178.89 (16)	N3—N2—C23—C24	78.6 (3)
C4—C5—C6—C7	−0.6 (3)	N1—N2—C23—C28	77.0 (2)
C5—C6—C7—C8	0.1 (4)	N3—N2—C23—C28	−107.2 (2)
C6—C7—C8—C9	−0.1 (4)	C28—C23—C24—C25	−1.2 (3)
C7—C8—C9—C4	0.6 (4)	N2—C23—C24—C25	172.68 (18)
C5—C4—C9—C8	−1.1 (3)	C23—C24—C25—C26	0.6 (3)
P1—C4—C9—C8	−178.98 (18)	C24—C25—C26—C27	0.2 (3)
C16—P1—C10—C11	130.83 (19)	C25—C26—C27—C28	−0.4 (4)
C4—P1—C10—C11	8.3 (2)	C24—C23—C28—C27	1.0 (3)
C1—P1—C10—C11	−108.0 (2)	N2—C23—C28—C27	−172.92 (19)
C16—P1—C10—C15	−50.06 (19)	C26—C27—C28—C23	−0.2 (4)
C4—P1—C10—C15	−172.54 (17)	N4—N3—C29—C34	−90.4 (2)
C1—P1—C10—C15	71.13 (19)	N2—N3—C29—C34	86.6 (2)
C15—C10—C11—C12	0.7 (4)	N4—N3—C29—C30	87.2 (2)
P1—C10—C11—C12	179.8 (2)	N2—N3—C29—C30	−95.8 (2)
C10—C11—C12—C13	−0.5 (4)	C34—C29—C30—C31	0.2 (3)
C11—C12—C13—C14	−0.4 (5)	N3—C29—C30—C31	−177.20 (19)
C12—C13—C14—C15	1.1 (4)	C29—C30—C31—C32	0.5 (4)
C13—C14—C15—C10	−1.0 (4)	C30—C31—C32—C33	−0.6 (4)
C11—C10—C15—C14	0.0 (3)	C31—C32—C33—C34	0.1 (4)
P1—C10—C15—C14	−179.11 (18)	C30—C29—C34—C33	−0.8 (4)
C10—P1—C16—C21	121.13 (19)	N3—C29—C34—C33	176.7 (2)
C4—P1—C16—C21	−118.17 (18)	C32—C33—C34—C29	0.6 (4)
C1—P1—C16—C21	4.0 (2)	N1—C22—C35—C40	6.5 (3)
C10—P1—C16—C17	−54.18 (19)	N4—C22—C35—C40	−175.14 (18)
C4—P1—C16—C17	66.53 (19)	N1—C22—C35—C36	−173.36 (19)
C1—P1—C16—C17	−171.35 (16)	N4—C22—C35—C36	5.0 (3)
C21—C16—C17—C18	1.1 (3)	C40—C35—C36—C37	−1.3 (3)
P1—C16—C17—C18	176.46 (18)	C22—C35—C36—C37	178.6 (2)
C16—C17—C18—C19	−1.5 (4)	C35—C36—C37—C38	−0.3 (4)
C17—C18—C19—C20	0.9 (4)	C36—C37—C38—C39	1.7 (4)
C18—C19—C20—C21	0.1 (4)	C37—C38—C39—C40	−1.6 (4)
C17—C16—C21—C20	−0.1 (3)	C38—C39—C40—C35	0.0 (3)
P1—C16—C21—C20	−175.23 (19)	C36—C35—C40—C39	1.4 (3)
C19—C20—C21—C16	−0.5 (4)	C22—C35—C40—C39	−178.46 (19)
C22—N1—N2—N3	−0.02 (19)		

### Hydrogen-bond geometry (Å, º)

**Table:**

*D*—H···*A*	*D*—H	H···*A*	*D*···*A*	*D*—H···*A*
C1—H1*A*···Cl2^i^	0.99	2.54	3.363 (2)	141
C1—H1*B*···Cl2^ii^	0.99	2.69	3.662 (2)	168
C3—H3*B*···Cl3^ii^	0.98	2.74	3.714 (2)	171
C3—H3*C*···Cl2^i^	0.98	2.90	3.630 (2)	132
C9—H9···Cl2^ii^	0.95	2.97	3.917 (2)	172
C24—H24···Cl3^ii^	0.95	2.99	3.783 (2)	142
C28—H28···Cl1	0.95	2.72	3.605 (2)	156
C30—H30···Cl3^ii^	0.95	2.87	3.683 (2)	144
C30—H30···Cl4^ii^	0.95	2.85	3.630 (2)	140

Symmetry codes: (i) −*x*+1/2, *y*−1/2, −*z*+3/2; (ii) *x*+1/2, −*y*+3/2, *z*+1/2.

## References

[bb1] Al-Ktaifani, M. & Rukiah, M. (2012). *Acta Cryst.* C**68**, m246–m250. 10.1107/S0108270112031885 22935491

[bb2] Bruker. (2015). *APEX2*, *SAINT* and *XP*. Bruker–Nonius AXS Inc., Madison, Wisconsin, USA.

[bb3] Clay, R., Murray-Rust, J. & Murray-Rust, P. (1975). *Acta Cryst.* B**31**, 289–290.

[bb4] Diop, T., Diop, L., Kučeráková, M. & Dušek, M. (2013). *Acta Cryst.* E**69**, o303. 10.1107/S1600536813002110 PMC3569822 23424568

[bb5] Diop, M. B., Diop, L. & Oliver, A. G. (2015). *Acta Cryst.* E**71**, m209–m210. 10.1107/S2056989015019180 PMC4719837 26870428

[bb6] Elangovan, A., Thamaraichelvan, A., Ramu, A., Athimoolam, S. & Natarajan, S. (2007). *Acta Cryst.* E**63**, m224–m226.

[bb7] Groom, C. R., Bruno, I. J., Lightfoot, M. P. & Ward, S. C. (2016). *Acta Cryst.* B**72**, 171–179. 10.1107/S2052520616003954 PMC4822653 27048719

[bb8] Haddad, S. F. & Al-Far, R. H. (2008). *J. Chem. Crystallogr.* **38**, 663–669.

[bb9] Krause, L., Herbst-Irmer, R., Sheldrick, G. M. & Stalke, D. (2015). *J. Appl. Cryst.* **48**, 3–10. 10.1107/S1600576714022985 PMC4453166 26089746

[bb10] Laus, G., Kahlenberg, V. & Schottenberger, H. (2015). *Acta Cryst.* E**71**, m110–m111. 10.1107/S2056989015006799 PMC4420035 25995889

[bb11] Sheldrick, G. M. (2015*a*). *Acta Cryst.* A**71**, 3–8.

[bb12] Sheldrick, G. M. (2015*b*). *Acta Cryst.* C**71**, 3–8.

[bb13] Wei, M. & Willett, R. D. (2002). *J. Chem. Crystallogr.* **32**, 439–445.

[bb14] Wikaira, J. L., Landee, C. P., Ludy, S. J. & Turnbull, M. M. (2013). *Polyhedron*, **52**, 770–780.

[bb15] Zhang, S.-F., Yang, X.-G., Liu, Z., Li, W.-H. & Hou, B.-R. (2007). *Acta Cryst.* E**63**, m1583.

